# Endocannabinoid System as a Promising Therapeutic Target in Inflammatory Bowel Disease – A Systematic Review

**DOI:** 10.3389/fimmu.2021.790803

**Published:** 2021-12-22

**Authors:** Szymon Hryhorowicz, Marta Kaczmarek-Ryś, Aleksandra Zielińska, Rodney J. Scott, Ryszard Słomski, Andrzej Pławski

**Affiliations:** ^1^ Institute of Human Genetics, Polish Academy of Sciences, Poznań, Poland; ^2^ Discipline of Medical Genetics and Centre for Information-Based Medicine, The University of Newcastle and Hunter Medical Research Institute, Newcastle, NSW, Australia; ^3^ Division of Molecular Medicine, New South Wales Health Pathology North, Newcastle, NSW, Australia

**Keywords:** inflammatory bowel disease, the endocannabinoid system, cannabinoid receptor, cannabis, ulcerative colitis, Crohn's disease

## Abstract

Inflammatory bowel disease (IBD) is a general term used to describe a group of chronic inflammatory conditions of the gastrointestinal tract of unknown etiology, including two primary forms: Crohn’s disease (CD) and ulcerative colitis (UC). The endocannabinoid system (ECS) plays an important role in modulating many physiological processes including intestinal homeostasis, modulation of gastrointestinal motility, visceral sensation, or immunomodulation of inflammation in IBD. It consists of cannabinoid receptors (CB1 and CB2), transporters for cellular uptake of endocannabinoid ligands, endogenous bioactive lipids (Anandamide and 2-arachidonoylglycerol), and the enzymes responsible for their synthesis and degradation (fatty acid amide hydrolase and monoacylglycerol lipase), the manipulation of which through agonists and antagonists of the system, shows a potential therapeutic role for ECS in inflammatory bowel disease. This review summarizes the role of ECS components on intestinal inflammation, suggesting the advantages of cannabinoid-based therapies in inflammatory bowel disease.

## Introduction

Inflammatory bowel disease (IBD) constitutes a group of chronic, relapsing, and incurable diseases of unknown etiology affecting the gastrointestinal (GI) tract that ultimately leads to the destruction of intestinal tissues and dramatically decreases the quality of affected patients’ life. Urbanization, improved sanitary conditions, increased use of antibiotics, a modern diet, the pace of life, and the associated stress in highly developed societies contribute to the alarmingly increasing number of IBD cases (1). At a clinical level, two major subtypes of IBD have been defined: Crohn’s disease (CD), which potentially affects any part of the gastrointestinal tract from the mouth to the anus, and ulcerative colitis (UC), an inflammatory condition limited to the colonic mucosa. The etiopathogenesis of IBD is still being debated. It has been postulated that multiple factors that include genetic susceptibility, the microbiome, environmental and immunological factors are involved in developing IBD that interact together resulting in the dysregulation of the intestinal tract immune system (2, 3). This dysregulation is the subject of intensive research to understand disease etiology and find novel treatments. Even though pharmacological treatment with aminosalicylates, glucocorticoids, immunosuppressive drugs, and biological agents is well-developed, all indications for the use of particular types of therapy are associated with the course of the disease and how patients tolerate treatment.

The use of pharmacological agents is associated with adverse side effects, which is particularly difficult due to long-term administration in the case of IBD treatment since more than half of IBD patients are chronically affected and hence eligible for long-term immunosuppressive treatment (4). Current immunosuppressive therapies have many long-term risks, including cancer, loss of immune tolerance, and low bone density. These negative consequences underscore the considerable demand for the development of highly innovative therapies for IBD (5).

The endocannabinoid system (ECS) has been recognized to play an essential role in maintaining gut homeostasis, regulation of intestinal function, feeding behavior, pain, intestinal inflammation, immune function, and neuroprotection (6). Since it quickly responds to disturbances by *de novo* synthesis of its effector molecules, it appears to be a new promising therapeutic target for IBD treatment. Even though the underlying biological mechanisms need to be clarified, these functions seem to be associated with the EC system’s capability to inhibit immune cell proliferation and cytokines, reactive oxygen species (ROS), and nitric oxide release (7). This review focuses on the role of type 1 and type 2 cannabinoid receptors and novel components of the so-called endocannabinoidome in IBD, describing their physiological and molecular functions and the adequacy of cannabinoid-based therapies in chronic IBD.

## ECS Role in Maintaining Immune System Homeostasis

The endocannabinoid system (ECS) is evolutionary stable (8, 9) which means it has been highly preserved in evolution for over 600 million years (9). Highly selective anandamide binding sites have been found in invertebrate immunocytes and microglia and are widely described in nearly all human tissues. The presence of the cannabinoid receptor in evolutionarily diverse organisms demonstrates that this ubiquitous signaling system has been conserved over many millions of years, serving multiple physiological roles, including gastrointestinal function regulation (8, 10). Cannabinoid receptor activation in the gut inhibits peristalsis, gastric acid secretion and increases food intake. It has been shown that ECS dysfunction may play a role in intestinal disorders including IBD, irritable bowel syndrome, and obesity (3, 11–13).

The ECS is composed of cannabinoid receptors: type 1 (CB1) and type 2 (CB2) - which are the main therapeutic targets of this system, endogenous ligands for the cannabinoid receptors such as anandamide (AEA) and 2-arachidonoylglycerol (2-AG), enzymes involved in their synthesis (diacylglycerol lipase DAGL and, *N*-acylphosphatidyl-ethanolamine phospholipase NAPE-PLD), cellular uptake and degradation (fatty acid amide hydrolase (FAAH) and monoacylglycerol lipase (MGL)), and lipid endocannabinoids such as oleoyl- and palmitoyl-ethanolamide (OEA and PEA) (8, 14–17). This complex signaling system, the so-called endocannabinoidome, is mainly concerned with the symptoms of commonly occurring neuropsychiatric disorders. As a key factor in the control of affective and cognitive functions and their pathological alterations, it also has a significant role in the microbiota-gut-brain axis (18, 19).

### Classical Cannabinoid Receptors

The two most commonly described cannabinoid receptors found in mammalian tissues are G-protein-coupled CB1 and CB2 receptors (20). The CB1 receptors are densely expressed in the brain, GI tract, neuronal tissues, central nervous system and where they mediate neurobehavioral effects that influence neurotransmitter release at axonic terminals, restoring, for example, the levels of interleukin 1 beta (IL-1β) and cyclooxygenase-2 (COX2) after inflammatory stimuli, as reported in rat studies (21). The CB2 receptor appears mostly on peripheral immune cells, such as B lymphocytes, macrophages, mast cells, natural killer cells, lymphatic organs, spleen, tonsils, and thymus (22). However, these receptors occur throughout the human body and can be found in almost all organs (23).

Determination of CB1 and CB2 receptors’ role in IBD was mainly confirmed by increased expression in an induced mouse model of colitis by dextran sulfate sodium (DSS) or increased mRNA expression in dinitrobenzene sulfonic acid (DNBS) induced colitis (24). CB1 receptor expression was also observed in models of croton oil-induced jejunal inflammation (25) or mustard oil-induced colitis (myenteric ganglia, endothelium) (26). High CB2 receptor expression is also observed in chemically induced colitis, mustard oil-induced colitis, and DSS (26). Numerous studies have shown that activation of CB1/CB2 receptors in CB1-deficient protects against experimental colitis in mice (27–29) in contrast to CB1-, CB2- deficient mice which had more severe IBD course than wild-type mice with induced inflammation. Mice lacking the CB1-, CB2-receptor or both receptors showed aggravation of inflammation in the model of TNBS colitis (30) which suggest that the endocannabinoid system may have tonic inhibitory effects on inflammatory responses in the colon (28).

CB1 receptors were found in the myenteric plexus (responsible for motor control of the GI tract) and submucosal plexus (responsible for secretomotor and vasomotor actions of the gut), and their locations in the gut ascertain their functions (31) CB2 receptors have also been identified in the immune cells, such as plasma cells and macrophages, in the GI tract’s lamina propria (32), in peripheral nerve terminals (33), and healthy and inflamed colonic mucosa (34).

The role of cannabinoid receptor antagonists and agonists is therefore not without significance in inflammatory processes. It appears that CB1 and CB2 receptors are expressed in practically all types of immune cells (T cells, B cells, NK cells, Dendritic cells, Macrophages, Neutrophils, Mast cells), implying that the inflammatory activity of these cells is regulated through the endocannabinoid activation (15–17), however showing some differences in downstream effects. Their activation may have similar effects by reducing VEGF-A secretion in human lung macrophages and (35) opposite effects e.g. reactive oxygen species (ROS) production and macrophage polarization (36). CB1 activation increases ROS and TNF-α production while CB2 activation inhibits these effects. Furthermore, CB1 induces macrophage polarization towards the M1 phenotype (37, 38) while CB2 activation changes the macrophages’ polarization *in vivo*. It has been suggested that CB2 agonists can improve IBD by regulating macrophage polarization as demonstrated by the CB2 receptor agonist (JWH-133), which significantly reduced the level of M1 markers (TNF-α, IL-1β, and IL-12) *in vitro*. Furthermore, the CB2 receptor agonist (JWH-133) enhanced the polarization of M2 macrophages induced by IL-4 by increasing the levels of Arg1, Mrc2 and Mgl1 which suggests that CB2 directly inhibits M1 phenotype macrophages and promotes macrophages to enter the M2 phenotype (39). In turn, CB2 activation by AEA reduces proliferation. It inhibits the release of proinflammatory cytokines from primary T cells indicating an anti-inflammatory effect of CB2 (40) while the CB2 receptor antagonist SR14452 blocked these effects (41). It appears that CB1/CB2 antagonists increased inflammation while endocannabinoids or synthetic agonists can have a protective effect. The CB1 receptor agonist ACEA was shown to alleviate inflammation in models of DSS-induced inflammation (26) and the cannabinoid agonist HU-210 protected against DNBS-induced colitis (24). Synthetic WIN 55, 212-2 was also found to protect mice against trinitrobenzene sulfonic acid (TNBS)-induced colitis and DSS-induced colitis, with simultaneous p38/Mk2 pro-inflammatory pathway blockade (24, 42). Nonetheless, this model does not reflect the human inflammatory cascade.

Similar conclusions were drawn from a study of oral treatment with α,β-amyrin, a pentacyclic triterpene, an organic compound from the terpene group, which reduced persistent inflammatory and the production of colonic pro-inflammatory mediators such as tumor necrosis factor (TNF)-α, interleukin (IL)-1β and keratinocyte-derived chemokine (CXCL1/KC) by direct activation of the CB1 and CB2 cannabinoid receptors (43, 44). It has been suggested that the anti-inflammatory effects of α,β-amyrin may be partially dependent on the interaction of CB1 and CB2 cannabinoid-mediated mechanisms (44). Earlier reports indicated that this compound is most likely involved in the inhibition of endocannabinoid hydrolases (45). Additionally α,β-amyrin inhibited the expression of adhesion molecule mRNA expression for ICAM-1, VCAM-1, PECAM-1, β(2)-integrin, proliferation marker Ki67, macrophage molecule CD68 and adhesion molecule P-selectin leading to an alleviation of DSS-induced colitis. In contrast, systemic administration of the selective CB1 receptor antagonist AM251 partially but significantly reversed the effect of α,β-amyrin, which in turn could not be confirmed for the pharmacological blockade of the CB2 receptor with AM630, a selective CB2 receptor agonist, as this did not affect inflammation (43). For the CB2 receptor, the agonist JWH-133 inhibited the pro-inflammatory cytokine IL-12p40 *in vitro* (46). Furthermore, a CB2 agonist, JWH-133 attenuated inflammation after chronic colitis in IL-10-/-mice by inducing apoptosis in activated T cells, both *in vitro* and *in vivo* (47). Similar observations were noted in a TNSB-induced colitis model which suggests a key role for CB2 receptors in ameliorating intestinal inflammation in mice and further highlights that CB2 receptor knockout seems crucial in regulating colitis (28, 48, 49).

The CB2 receptor pathway has also been shown to be potently modulatory for atypical cannabinoids. Palmitoylethanolamide (PEA) and cannabigerol (CBG) were reported to have beneficial effects in a DNBS-induced colitis model. PEA has been shown to improve the course of experimental colitis in mice by reducing the weight/length ratio of inflamed colonic tissue, which is considered a reliable indicator of the severity of the extent of the inflammatory response. PAE stimulated colonic cell proliferation and intestinal permeability, and increased the expression of TRPV1 and CB1 receptors in the colon. In contrast, the effects of PEA were abolished by CB2, TRPV. GPR55 and PPARα receptor antagonists (50). Furthermore, CBG enhanced glandular regeneration, reduced granulocyte infiltration into the mucosa and submucosa, and restored intestinal epithelial integrity. Moreover, it reduced the diffusion of Ki-67, a prognostic colon cancer marker (51).

CB1 signaling mediates neuromodulatory function. The endocannabinoid system regulates pain processing through activated CB1. Their activation by both exogenous cannabinoids and arachidonic acid-derived endocannabinoids also leads to increased appetite, promotion of food intake and weight gain (52–54). CB1 also inhibits the activation of N- and P/Q type intracellular calcium channels, decreasing calcium release, but activates inward-rectifying potassium and potassium-A channels and mitogen-activated protein kinase (55, 56). When cannabinoids bind to the prejunctional CB1 receptors, reduced excitatory neurotransmission causes decreased gut motility and secretion (57). The activation of CB1 receptors helps control emesis by reducing excitatory neurotransmitters such as glutamate in the dorsal vagal complex (58, 59), whereby simultaneous activation of CB1 and CB2 receptors produces anti-emetic, anti-motility, and anti-inflammatory effects through inhibition of adenylyl cyclase with the reduced cAMP formation (Gi/o coupled), thus blocking neurotransmitter release from a presynaptic neuron by CB1 and pro-inflammatory cytokine release by CB2 (32, 60).

Cannabinoid receptor type 2 is responsible for regulating B cell and T cell differentiation and balancing of Th1 pro-inflammatory to Th2 anti-inflammatory cytokines. Besides, in macrophages, CB2 stimulation inhibits an increase in and a release of pro-inflammatory factors TNF-a, IL-12p40, and IL-1, which promote the inflammatory response (61) Kapellos et al. (2019) report revealed that endocannabinoid signaling through inhibits neutrophil recruitment during the acute inflammatory response (62). Immunohistological studies of CB1 and CB2 receptors, in IBD patients’ colonic tissues, showed high CB1 and CB2 immunoreactivity of the epithelium in the acute phase, whereas the healthy human colon epithelium did not show higher CB2 activity. This observation may provide evidence that CB2 receptors play a significant role in the disease course and mediate disease immunomodulatory activity (34). This finding is also supported by studies showing that functional variants of CB2 contribute to an increased risk of IBD or chronic inflammatory disease of the small intestine - celiac disease (63, 64). The results indicate that the CB2 receptor plays a pivotal role in the pathogenesis of IBD (5) and suggest it may represent a molecular target for pharmacological modulation of immune components.

Cannabinoid receptors are most often described in the context of anti-inflammatory effects *in vivo* (65–67). Henshaw et al. have described a comprehensive insight into cannabinoid-mediated pro-and anti-inflammatory cytokine responses in preclinical *in vivo* studies. Moreover, they have indicated that the combination of cannabinoids, namely CBD+CBG, and CBD+THC can show the most effective anti-inflammatory effect *in vivo*. This information may be essential in the further human clinical trials of cannabinoids based on using CBD and CBG as the inhibitors of inflammation across a range of pathophysiological processes (66).

### Novel Cannabinoid Receptors

The ECS’s involvement in various mechanisms and processes in the gastrointestinal physiology, such as intestinal motility, secretion, epithelial barrier integrity, intestinal inflammation, or immune modulation through signaling pathways (68), as well as its complex pharmacology, suggests that other receptors may be potentially missing members of the ECS (69). More recently, it has become apparent that endocannabinoids do not exclusively bind to CB1 and CB2 receptors but also activate or inhibit so-called orphan G-protein-coupled receptors. The close phylogenetic relationship and the high percentage of structural motif homology with cannabinoid receptors indicate that orphan receptors seem to play a crucial role. These include GPR3, GPR6, and GPR12, which show more than 60% similarity (70); GPR40, GPR41, GPR43, GPR55, GPR84, and GPR120 with suggested modulatory role in the course of chronic inflammatory diseases (71).

Epidemiological studies show an increasing role of the endocannabinoid system (ECS) in chronic inflammatory diseases, including diabetes mellitus, arteriosclerosis, and IBD. ECS and the cannabinoid receptors contribute to modulating regulation of inflammation immune response, cell proliferation, gastrointestinal motility, visceral sensation, gut homeostasis, and intestinal barrier maintenance (3, 72). The loss of intestinal epithelium integrity and dysfunction of the innate immune system plays a key pathogenetic role in IBD chronic mucosal inflammation, particularly in the small intestine and colon. Mucosal inflammation leads to alterations in intestinal transit, increases in protons, lactate, and bicarbonate products, as well as pro-inflammatory cytokines, chemokines, and adhesion molecules, and changes in the uptake of short-chain fatty acids - which significantly affect the balance between pro and anti-inflammatory responses and may explain the abnormal changes in pH in the intestinal lumen (3, 73, 74). In IBD, the microenvironment of the intestine is characterized by pH levels between 7.0 or even 6.0 (75, 76). Thus, it seems that identifying signaling pathways and processes determining cellular activity and function in an acidic microenvironment would be of crucial importance. It appears that orphan receptors from the G-protein-coupled receptor family, as so-called proton-sensing GPCRs, play an essential role in these processes. It turns out that GPCRs recognize protons within histidine residues and lead to inflammation and immune response. Moreover, GPR4, GPR65, GPR68, and GPR132 are responsible for leukocyte promotion and adhesion, expression of inflammatory genes, inflammatory responses, and the maintenance of epithelial barrier function, which suggests a crucial role in IBD (3, 76).

More attention should be paid to the GPR55 receptor, which was previously suggested to be a third cannabinoid receptor, through its possible activation by endocannabinoids and synthetic cannabinoid ligands (77–79). However, its classification as a cannabinoid receptor is not entirely straightforward due to its pharmacology, signaling, and cellular function. Although it has low homology and phylogenetic distinctiveness to CB1 and CB2 receptors (sequence identity at the level of 13,5 and 14,4%, respectively) and lack of classic cannabinoid binding sites, it shows high affinity for cannabinoids and cannabinoid ligands such as Δ^9^-THC, 2-arachidonoylglycerol, anandamide, or rimonabant, which is independent of CB1 and CB2 receptors and their signaling pathways (80, 81).

The first evidence for an important pharmacological role for GPR55 was described in the GlaxoSmithKline patent (submitted by Brown and Wise, 2001) that documented GPR55 expression mediated by the CB1 antagonist AM251 (82). Since then, many reports describing the pharmacology of GPR55 and its interaction with various ligands in different cell types have been published. These results, however, were not conclusive and in some cases contradictory (78, 80, 82–85) GPR55 is predominantly coupled to G13 and Gq, which results in the activation of the RhoA/ROCK and PLC/Ca^2+^ signaling pathway, which may be involved in different cellular responses than just *via* classical cannabinoid receptors (86). It has been suggested that due to its mediation of pro-inflammatory cytokine release and activation of gut neurons in the gastrointestinal tract in rodents, GPR55 may play an essential role in the induction of intestinal inflammation (87) which has been reported by Lin et al. who showed that GPR55 is present throughout virtually all of the rat intestine and is up-regulated *via* LPS-induced inflammation (88). This up-regulation has also been confirmed in IBD patients, where mRNA expression levels and colonic GPR55 concentrations, were significantly higher than in controls (89). Interestingly, GPR55-/- knockout mice in a DSS-induced mouse model of intestinal inflammation revealed rodents had less intensive response than wild-type mice, clearly indicating a pro-inflammatory role for GPR55 in intestinal inflammation. This effect is reinforced by the fact that treatment with the GPR55 antagonist CID16020046 significantly reduced the expression of pro-inflammatory cytokines and inhibited leukocyte activation and accumulation, which are characteristic features of IBD (90). In contrast, O-1602 (a GPR55 agonist) showed a protective effect against experimentally induced colitis, reducing inflammation and inhibiting neutrophil migration even in CB1/CB2 and GPR55 knockout mice (29). Thus, evidence accumulate that indicatings that pharmacological blockade of GPR55 prevents the development and progression of intestinal inflammation, and this way could be an attractive target in the treatment of IBD. Nevertheless, the ambiguous function of this receptor and the lack of knowledge about GPR55 pathways in inflammatory processes do not yet allow substantial conclusions to be drawn.

Some non-cannabinoid receptors, such as TRP (transient receptor potential channel) TRPV1, TRPV2, TRPV3, TRPV4, TRPA1, and TRPM8 should also be considered as potential therapeutic targets. TRPV1 agonists (capsaicin, resiniferatoxin, SA13353), may induce an anti-inflammatory effect during colitis inflammation (91–93). In induced small intestine inflammation in rodents, anandamide levels were elevated and influenced inflammation exacerbation through TRPV1 receptor activation (94). It appears that TRPV1 has a protective function against inflammatory changes by synthesizing and/or releasing endovanilloids, and that its activation is a crucial initial step in the immune response. In the human colon, vanilloid receptor TRPV1 is overexpressed both in afferent nerve terminals and in epithelial cells during inflammation. In the past years, pharmacological experiments using TRPV1 agonists and antagonists revealed that TRPV1 receptors may play proinflammatory and protective roles in the gastrointestinal tract. Genetic approaches were used to define the role of TRPV1 and analyze the effects of dinitrobenzene sulfonic acid (DNBS)-induced colitis in TRPV1-deficient (TRPV1-/-) mice. Intrarectal infusion of DNBS induced increased inflammation in TRPV1−/− mice compared to wild-type littermates (TRPV1+/+) as evaluated by macroscopic scoring and myeloperoxidase assays. This finding indicates that TRPV1 receptors are required for the protection within sensory pathways that regulate the response following the initiation of colonic inflammation. Electrophysiological recordings from circular smooth-muscle cells, performed 8 and 24 h after DNBS treatment, revealed strong spontaneous oscillatory action potentials in TRPV1−/− but not in TRPV1+/+ colons, indicating an early TRPV1-mediated control of inflammation-induced irritation of smooth-muscle activities. These unexpected results suggest that TRPV1 receptors mediate endogenous protection against experimentally induced colonic inflammation. Given the reports of reduced protection against DNBS-induced colitis by deletion of both CB1 (94) and TRPV1 receptors (95), it appears that the action of anandamide on both receptors could mediate protection against colitis. Enhancement of the activity of anandamide (or other endogenous CB1 and TRPV1 receptor ligands) may represent a promising therapeutic target for the effective control of excessive inflammatory responses in the colon. It is also worth bearing in mind the peroxisome proliferator-activated receptors (PPAR- α, γ, and δ), 5HT3 receptors, as well as potassium channels, oleoylethanolamide, and palmitoylethanolamide together with TRPV1, are called novel components of ECS (defined as endocannabinoidome) (15, 96, 97). However, given the complexity and wide range of functions of the individual components of the ECS and the fact that there are still many unknowns, it remains difficult to define its precise boundaries unambiguously.

### Main Endogenous Agonists of CB Receptors

Endocannabinoids include amides and esters of long-chain polyunsaturated fatty acids, of which anandamide (AEA) and 2-arachidonoylglycerol (2-AG) are the main endogenous agonists of CB1 and CB2 (98) and have the most potent biological activities. AEA has a low affinity for CB2 but a high affinity for CB1, while 2-AG binds well to both receptor types (98, 99). They are synthesized on-demand and rapidly degraded according to the needs of cell membrane phospholipids and act on specific cannabinoid receptors in an autocrine/paracrine manner. Both agonists are derivatives of arachidonic acid (AA), delivered from membrane-bound lipid precursors and have similar three-dimensional structures but are synthesized and degraded through different pathways. The two agonists are involved in several pathological conditions of the small and large intestine (12, 24, 100, 101).

In the context of inflammatory processes, AEA protects the gut from inflammation by reducing transcription and secretion of interleukin (IL)-6, IL-12, tumor necrosis factor (TNF)-α, and interferon-α (102, 103). It is a partial agonist for both CB1 and CB2 receptors and can be hydrolyzed to AA and ethanolamine by fatty acid amide hydrolase (FAAH) (67, 104). In addition, AEA is responsible for inducing lymphocyte apoptosis and inhibiting neutrophil recruitment at the site of inflammation (40). Upon binding with CB2, AEA inhibits the Th1 response through IFN-γ, and Th17 response, while suppressing T cell activity which may suggest a potential down-stream effect of AEA in modulating communication between immune cells such as T and B lymphocytes, macrophages and neutrophils, activation of which is associated with immune diseases (40). Animal studies have shown that the activation of CB2 receptors produces peripheral antinociception and reduced inflammatory edema (105). Acharya et al. showed that the endogenous cannabinoid AEA’s engagement is a major participant in ensuring tolerance in the gut through maintenance or differentiation of the well-known immune regulatory CX3CR1hi Mϕ population and by mediating expansion of the immunosuppressive Tr1 cells (8),. Moreover, Sabatino and colleagues showed that the non-hydrolyzable AEA analog methanandamide (MAEA) was found to have anti-inflammatory effects *in vitro* and *ex vivo* as evidenced by a reduction in pro-inflammatory cytokine levels in patients with IBD (103). Interestingly anandamide (AEA) and oleoylethanolamide (OEA) levels were elevated in the plasma of ulcerative colitis and Crohn’s disease patients, while 2-arachidonoylglycerol (2-AG) was elevated in UC but not in CD patients (68). Furthermore, increasing endogenous 2-AG levels (by treatment with MGL inhibitors and palmitoylethanolamide (PEA) and AEA proved that exogenous and endogenous cannabinoids can reduce gastrointestinal inflammation (106–108) which supports the theory that ECS is “triggered” by the inflammation to restore homeostasis (109). The results of Grill et al. clearly show that in colonic mucosal biopsies from UC patients compared to controls, there was a significant increase in AEA (but not 2-AG) and PEA, and in contrast, a significant 2-AG increase in CD patients (68), which is consistent with reports from other research groups (110, 111). Moreover gene expression studies in intestinal mucosal biopsies showed different profiles in CD and UC. In CD, increased gene expression was observed for the 2-AG synthesizing enzyme diacylglycerol lipase alpha. Most of the ECS transcripts were examined (*NAPE-PLD, DAGLalpha*, and *DAGLbeta*, *FAAH, MGLL, NAAA, ABHD6*) also showed this trend, in contrast to UC patients (68). D’Argenio confirmed that colitis is accompanied by increased levels of anandamide, but not 2-AG, in both rodents (colonic submucosa) and ulcerative colitis patients (mucosa) (112). Based on the reports of the protective effect of CB1 cannabinoid receptor stimulation, it is possible to speculate on possible new therapeutic strategies against IBD where CB1 receptor agonists and antagonists may exert a protective effect against inflammation. Thus, increasing endogenous levels of 2-arachidonoylglycerol, as a full agonist of these receptors, should reveal beneficial effects on colitis. The Belgian group demonstrated that increasing the 2-AG level *via* inhibition of monoacylglycerol lipase (MAGL) (responsible for 2-AG hydrolysis) by JZL184, resulted in an increase of 2-arachidonoylglycerol (113). Elevated 2-AG levels led to decreased expression of pro-inflammatory cytokines and inflammatory lesions at the macroscopic and histological levels. Interestingly, administration of selective CB1 (SR141716A) or CB2 (AM630) antagonists along with JZL184 nullified the protective effect of MAGL inhibition, thus demonstrating the involvement of both cannabinoid receptors (113). These findings may suggest an important protective role for 2-AG, which could provide some foundation for novel pharmacological intervention.

## Endocannabinoid System in IBD

**Figure 1:**
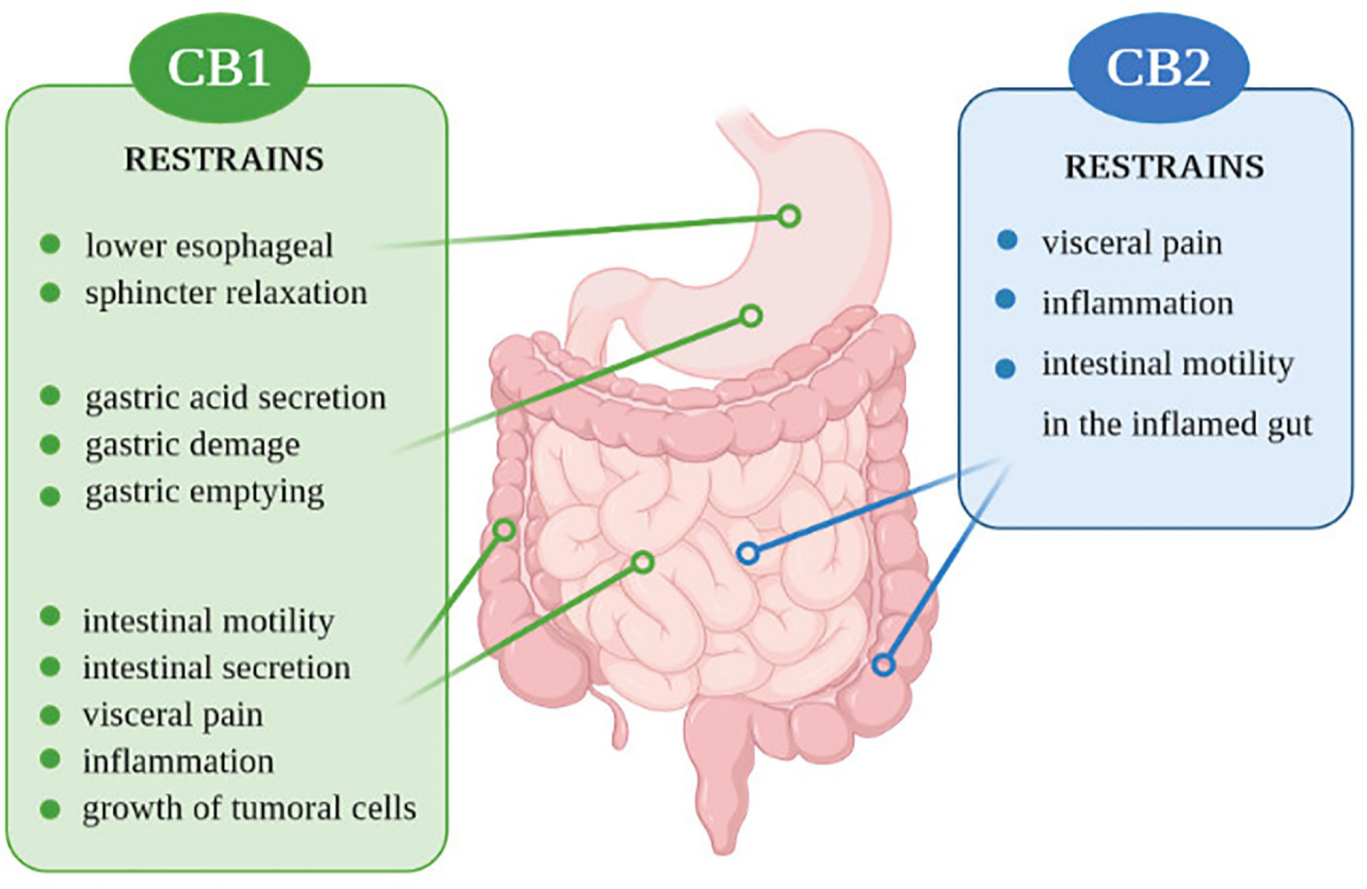
Action of CB1 and CB2 receptors in the digestive system.

Components of the ECS and the endocannabinoidome are involved in various GI tract mechanisms, starting on the regulation of food intake and satiation, nausea, vomiting, gastric secretion, gastroprotection, gut motility, visceral sensation, intestinal inflammation, maintenance of epithelial barrier integrity, and immune tolerance regulation (12, 114) (**Figure 1**). Thus, control of these mechanisms in the gastrointestinal tract may be important in preventing IBD.

Preclinical studies conducted in recent years have shown that cannabinoids play a strong protective function (15, 68, 115). Several *in vitro* studies have demonstrated that CB1 and CB2 receptor agonists reduced experimentally induced intestinal inflammation. At the same time, antagonists exacerbated inflammatory processes (24, 26, 116). There are only a few published reports concerning the expression of ECS members in mucosal tissue of IBD patients. So far, have been limited reports on the upregulation of CB1 (103) and CB2 receptors (28). Stintzing et al. revealed that the *CNR1* gene encoding the CB1 receptor displayed significant up-regulation, particularly in patients with CD (117). In 2009, Marquéz and collaborators reported differences in CB receptor protein levels and metabolic enzymes in. UC patients’ acute and quiescent colonic epithelial tissue. They found low NAPE-PLD levels and elevated DAGL-alpha and CB2 receptor levels in inflamed tissue, while quiescent tissue fragments had decreased CB1 expression. MAGL was elevated both in acute and quiescent tissue (118). A similar observation was made by Di Sabatino et al., who assessed enzymatic activity in the inflamed and uninflamed intestinal mucosa of UC and CD patients. They observed decreased activity of NAPE-PLD in inflammatory lesions and regular activity in non-inflamed tissue. Unfortunately, they did not examine DAGL enzymes, but they did investigate FAAH activity, which was increased in inflamed and decreased in unchanged mucosa. Regarding CB receptors levels, Di Sabatino et al. reported conflicting results compared to those of Marquéz et al.: CB1 levels were increased, while CB2 remained unchanged. They also assessed EC levels and found that 2-AG and PEA were stable in UC and CD inflamed and non-inflamed mucosa. However, they observed decreased AEA levels in inflamed tissue (103), compared to three other studies (112, 119, 120).

The most extensive and comprehensive research of ECS components in IBD patients is the latest study of Grill et al., which reports conflicting results to several previous findings (68). Grill et al. showed altered plasma levels of endocannabinoids in IBD and distinct transcript profiles in UC and CD. Using the NanoString hybridization method to assess mRNA from intestinal mucosal biopsies, the authors revealed that most CB and non-CB receptor transcripts, particularly CB1 and GPR119, were downregulated in IBD patients in comparison with healthy controls. Moreover, they also reported that AEA and OEA were increased of both UC and CD patients plasma, whereas 2-arachidonoylglycerol (2-AG) was elevated in patients with CD exclusively. They assumed that one explanation for the decrease CB_1_ gene expression could be downregulation due to high levels of its ligand, AEA, which could have caused the reduced expression. Concerning GPR55, low mRNA levels were detected in healthy tissue in a few epithelial cells and lymphocytes in the lamina propria. Biopsies of CD patients showed low mRNA signals in the lamina propria. In UC patients, GPR55 mRNA was generally low but slightly elevated in epithelial cells (68). Expression data for genes coding ECS proteins and the measured levels of various ECS components are summarized in **Table 1**, which shows how much remains to be investigated.

**Table 1:** The expression data for genes coding ECS proteins and the measured levels of various ECS components.

Authors	Grill et al., (68)		Di Sabatino et al., (103)		Stintzing et al., (117)		Marquez et al., (118)	Nicotra et al., (120)	D’Argenio et al., (112)	Darmani et al., (119)
**Sample type**	Inflamed intestinal mucosa/plasma		Inflamed intestinal mucosa		Colectomy specimens		Inflamed colonic tissue	Inflamed colonic mucosa	Biopsy from the rectum or the most inflamed area	Inflamed colonic mucosa
**Assessed material**	mRNA level/receptors ligands		protein level/enzymes activity		mRNA level		Protein level	Enzyme activity	–	Protein level
**Method**	NanoString hybridization method/mass spectrometry		HPLC electrospray ionization mass spectrometry/radiochromatographic method		qRT-PCR		Immunocytochemistry	Fluorometric quantification	Liquid chromatography-atmospheric pressure chemical ionization-mass spectrometry (LC-APCI-MS)	Mass spectrometry
**Patients**	**CU**	**CD**	**CU**	**CD**	**CU**	**CD**	**CU**	**CD**	**CU**	**UC**
**Receptors encoding genes (proteins):**										
*CNR1* (CB1)	decreased	decreased*	increased*	increased*	decreased*	increased*	unchanged	–	–	–
*CNR2* (CB2)	decreased	decreased	unchanged	unchanged	unchanged	unchanged	increased	–	–	–
*GPR18*	decreased	decreased	–	–	–	–	–	–	–	–
*GPR55*	decreased	decreased	–	–	–	–	–	–	–	–
*GPR119*	decreased	decreased*	–	–	–	–	–	–	–	–
*TRPV1*	increased	increased*	–	–	–	–	–	–	–	–
*PPARA*	decreased*	increased	–	–	–	–	–	–	–	–
*PPARG*	decreased	increased	–	–	–	–	–	–	–	–
*PPARD*	increased	increased*	–	–	–	–	–	–	–	–
**Genes for ligands synthesizing enzymes (enzymes):**										
*NAPE-PLD* (NAPE-PLD)	decreased*	increased	decreased*	decreased*	–	–	decreased*	–	–	–
*DAGLA* (DAGL-α)	increased	increased*	–	–	–	–	increased*	–	–	–
*DAGLB* (DAGL-β)	increased	increased	–	–	–	–	unchanged	increased*	–	–
**Genes for ligands degrading enzymes (enzyme**s):										
*FAAH* (FAAH)	decreased	increased	increased*	increased*	–	–	unchanged	–	–	–
*NAAA*	increased	increased	–	–	–	–	–	–	–	–
*MGLL*	decreased	increased	–	–	–	–	increased*	–	–	–
*ABHD6*	decreased*	increased	–	–	–	–	–	–	–	–
**Endogenous receptors ligands**										
AEA	increased*	increased*	decreased*	decreased*	–	–	–	–	increased*	increased*
2-AG	unchanged	increased*	unchanged	unchanged	–	–	–	–	slightly decreased	–
PEA	increased*	increased*	unchanged	unchanged	–	–	–	–	–	increased*
OEA	increased*	increased*			–	–	–	–	–	–

* - statistically relevant.

However, some studies have reported levels of endocannabinoidome members from animal models of IBD over the past decade. In the colitis model, the researchers found that CB1, CB2, and AEA were up-regulated. Simultaneously, the expression of AEA degrading enzyme: FAAH was decreased (79) which is the opposite of studies reported by Nicotra et al. on the actively inflamed mucosa of Crohn’s patients, which revealed that FAAH activity was significantly elevated when compared to non-inflamed colonic tissue (120). Ileal expression of CB1 and AEA was increased in actively inflamed TNF^∆ARE/+^ mice compared with controls. CB2 receptor mRNA was preferentially induced on regulatory T cells in TNF^∆ARE/+^ mice. These mice are characterized by a 69 bp deletion within the AU-rich element of the *TNF* gene, contributing to the overproduction of TNF, consequently leading to chronic colitis. They represent an excellent model of inflammation due to their similarities in pathogenesis and histology to human Crohn’s disease. Furthermore, GP-1a enhanced a Treg suppressive function with elevated IL-10 secretion, ameliorating ileitis in this research model (121).

Studies with animal models of colitis have shown that PEA degradation inhibition can significantly improve the inflammatory response of experimental colitis. Accordingly, THC and PEA’s oral administration can increase the anti-inflammatory impact on the intestinal tract (113). In a murine model of trinitrobenzene sulfonic acid-induced colitis, administration of CB2 agonists (JWh133 and AM1241) upregulated the CB2 receptors and reduced colonic inflammation (28). The use of cannabinoids prevents the onset of experimental colitis or minimizes its severity. Clinical studies described by Carvalho et al. (122) have investigated the effects of cannabinoid ligands or the effect of blocking their metabolizing enzymes on inflammation of the intestine and have shown significant promise at a preclinical level for IBD treatment (24, 123).

## Cannabinoids in IBD Treatment

Natural cannabinoids include those produced by plants, the phytocannabinoids and endocannabinoids that are synthesized by mammals. Production of synthetic cannabinoids (SCs) began in the early 2000s and is currently rapidly expanding, but at the same time leaving much concern about the SCs’ use and safety. SCs are often designed to have a higher affinity for their receptor and a longer-lasting effect than their natural analogs (124). Nowadays, there is a significant interest in treating IBD with highly efficient therapies, including all available and promising conventional methods. Most patients focus on alternative therapies, such as cannabis, to minimize persistent clinical symptoms associated with IBD. *Cannabis sativa* L has a complex chemical composition of over 500 different constituents that include: terpenes, carbohydrates, fatty acids, esters, amides, amines, phytosterols, and more than 100 phenolic compounds – cannabinoids (125–128). The analgesic and antinociceptive activity of selected cannabinoids have been proven in several human (116, 129, 130) and animal models (131–133) Significant growth in research aimed at understanding the physiological functions of cannabinoids was the identification of their two main components namely cannabidiol (CBD) and tetrahydrocannabinol (Δ⁹-THC) that can activate endogenous CB1 and CB2 receptors. THC and CBD have a varying affinity for cannabinoid receptors, with THC having a higher affinity for CB1 whereas, CBD does the opposite and has a very low affinity for both types of the cannabinoid receptor. CBD promotes the natural production of endogenous cannabinoids in an indirect or “circuitous” way (134). However, CBD is also considered a highly potent antagonist, which acts as a negative allosteric modulator of the CB1 receptor *via* alternating the orthosteric ligands effectiveness and potency while not activating a highly potent antagonist the receptor itself (135, 136). Furthermore, there is evidence that CBD may mitigate some of the effects of THC, potentially *via* indirect agonism, either by augmenting CB1 constitutional activity or endocannabinoid binding (137). Therefore, CBD will not produce psychoactive activity reactions in the brain and central nervous system as does THC but only promotes natural cell repair. However, it was reported that CBD could act as an antagonist at certain concentrations below which it binds to both CB1 and CB2 orthosteric sites (137, 138). Portland et al. showed that CBD binds to a distinct, allosteric site on CB1 receptors with a different function than the orthosteric site for 2‐AG and THC. This role of CBD as a negative allosteric modulator may explain its antipsychotic, anti‐epileptic, and antidepressant features and may be useful in the development of novel CB1 receptor‐selective drugs (136). CBD activates other various receptors and ion channels that have a multitude of positive impacts. When the receptor and CBD bind, downstream signaling pathways are activated, which result in protein kinase activity. The changes of PKA, PKC, RAF‐1, ERK, JNK, p38, and other molecules combined with cAMP activated PKA/cAMP channels. Cannabinoids also regulate the phosphorylation of different members of the mitogen-activated protein kinase (MAPK) family by binding with cannabinoid receptors, including extracellular signal-regulated kinases 1 and 2 (ERK1/2), p38, MAPK, and amino-terminal kinase, which result in the activation of MAPK signaling pathways, which orchestrate the corresponding response of the intestinal mucosal immune system (139).

CBD exerts its immunosuppressive effect *in vivo* and *in vitro* mainly by inducing apoptosis, inhibiting cell proliferation, inhibiting the production and activation of cytokines and chemokines (140). At the same time, CBD has been shown to reduce the activity of B cells, activate T cells (by increasing the level of apoptosis), and induce Treg cells to inhibit the production of cytokines, and ultimately prevent the inflammatory response *in vivo* (141).

CBD can promote the production of IL‐4 and IL‐10 related to Th2 by inhibiting the release of pro-inflammatory factors such as IL‐1, IL‐12, TNFα, and interferon (INF) ‐ G from monocytes in peripheral blood and intestinal tissues. Moreover, CBD inhibits interleukin-6, a proinflammatory cytokine produced by many cell types (142) and also can inhibit interleukin-8 production by activated B cells, which could have anti-inflammatory effect and potential therapeutic importance in immune disorders (143, 144) It also regulates the phenotypic differentiation of monocytes into M1 or M2 macrophages, the production of cytokines, chemokines, and other immune mediators, and inhibits dendritic cell markers such as MHCII CD86 and CD40 (145). Moreover, it reduces the levels of serum immunoglobulin, the number of B cells, and the levels of IgG and IgM immunoglobulins (146).

Although the therapeutic effect of CBD on IBD has been verified in experimental animal studies, no large-scale clinical trials have been carried out at present, and only the inflammatory relief in most IBD patients after taking cannabinoids has been observed.

The focus on the therapeutic potential of *Cannabis Sativa* L. containing different cannabinoids in IBD treatment has been continually rising over the past years. Although the few randomized controlled trials in patients with IBD have not yet confirmed the tremendous impact of cannabis in modulating inflammatory disease activity, the preclinical IBD models have already proven that cannabinoids may alleviate intestinal inflammation in experimental IBD models through their interaction with the ECS (147, 148).

Although it has been proposed that the influence of the ECS is helpful for the treatment in IBD, additional studies are repeatedly undertaken to confirm this hypothesis. It has already been shown using experimental models of rodent colitis that, e.g., cannabidiol, which has anti-inflammatory and immunomodulatory properties, can be used as an alternative agent in IBD (149). It is worth reinforcing that in 2015 epidemiological studies were conducted to define the number of patients with IBD who used cannabis to relieve their symptoms of disease. Despite the limited legality of cannabis use, based on available reports, it has been demonstrated that between 6.8 and 17.6% of IBD patients regularly use cannabinoids (149). These patients have explained that using cannabis helps reduce symptoms that include abdominal pain, nausea, diarrhea, or anorexia, and visibly improves their general well-being (150). Among the final reports from these studies it has been revealed that cannabis mainly alleviates abdominal pain (83.9%), abdominal cramping (76.8%), joint pain (48.2%), and diarrhea (28.6%) (147, 151). It is widely known that *Cannabis Sativa* L. is highly recommended for the treatment of chronic pain and a variety of neurological conditions, but is also promoted as an effective medicine in other gastrointestinal conditions, e.g., anorexia, emesis, abdominal pain, diarrhea, and diabetic gastroparesis (149). Therefore, the beneficial properties of cannabinoids that occur in cannabis, and their positive influence on IBD activity should be taken into account (147). According to Naftali et al., cannabis treatment is highly recommended in IBD patients since there is now evidence that the administration of cannabinoids may reduce the need for other medications and also the risk of surgery (152). Moreover, the studies have also indicated that the reduced disease activity that has been observed is associated with the cannabis use for IBD treatment (149, 152, 153).

In turn, the recent randomized, controlled trial carried out by Naftali et al. has assessed the safety of medical cannabis in an IBD population. Patients with moderate to severe Crohn’s disease have used CBD-THC oils for 8 weeks. As a result, both clinical and endoscopic outcomes have been determined (154). It is worth emphasising that some reports of cannabis use in IBD patients in the United States and Canada have shown that 15-20% of patients regularly use cannabis. Moreover, up to 40% used cannabis to reduce IBD symptoms, such as pain, diarrhea, and appetite enhancement (155, 156).

In 2011, a randomized controlled trial of medical cannabis for the treatment of Crohn’s disease in Israel (152) was undertaken, resulting in a reduced need for other medications and surgery for the cannabinoids-user patients. Patients (n=21) smoke twice daily cigarettes containing 115 mg of THC and then were compared to the placebo group. Consequently, 90% of patients taking cannabinoids have shown a decrease in their Crohn’s Disease Activity Index (CDAI). Additionally, 25% of patients could stop corticosteroid therapy. Besides notable enhancements in quality of life, pain scores, or appetite, there were no improvements in markers, i.e. C reactive protein (CRP) (147).

A second small study has shown that IBD patients (n=19), who were usually treated with steroids, thiopurines, or TNF antagonists, were randomized to receive 10 mg of oral CBD or placebo twice daily for 8 weeks. This study based on the oral formulation of cannabis has not shown any changes in IBD activity assessed by the CDAI or laboratory parameters between the treatment group and placebo (147).

In turn, the first randomized controlled trial regarding ulcerative colitis was associated with the safety and efficacy of CBD-enriched botanical extract. Patients (n=60) with different ulcerative colitis stages were randomized to receive a once-daily oral capsule containing 50 mg of CBD from botanical extract or placebo for 10 weeks. The final reports have shown that the CBD extract was not well tolerated (157).

According to Ahmed and Katz (158) cannabinoid’s use should be considered as a potential therapy for IBD, especially in patients with a severe IBD course, and resistant to traditional medicines. The therapeutic anti-inflammatory effect of cannabinoids in IBD has not been precisely determined yet (159). It is thought that they can also hide many other debilitating symptoms. Therefore there is a need for double-blind, randomized, placebo-controlled trials using serial inflammatory markers, biopsy findings, and endoscopic disease severity to demonstrate objective improvement in IBD (158, 160).

Naftali et al. have determined the effects of licensed cannabis use among patients with IBD. The authors have shown that both THC (21 mg/day) and CBD (170 mg/day) was associated with clinical improvement (161). Although researchers have shown that cannabis has therapeutic potential in IBD more studies are needed to confirm the health benefits of the various cannabis compounds. These effects can be assessed in randomized placebo-controlled clinical trials, where it is entirely possible to confirm the potential of cannabis treatment in IBD (162). As Cannabis sativa L. is a promising source of phytocannabinoids, further studies have been launched to identify other plants secreting CBs. The CB1 ligands were found in *Daucus carota L*., *Piper methysticum*, and *Heliopsis helianthoides L* (163). Other plants can produce cannabinoid-like molecules such as perrottetinene extracted from *Radula perrottetii*, or anandamide extracted from some bryophytes. Several other secondary metabolites - so-called cannabimimetic-can also act as ECS receptor agonists or antagonists or ECS enzyme inhibitors (164).

An important aspect is the use of synthetic cannabinoids in IBD treatment. Until now, the US Food and Drug Administration (FDA) has not approved cannabis, cannabis-derived, or cannabidiol (CBD) products currently available on the market for the treatment of IBD. However, the agency has accredited one cannabis-derived drug: Epidiolex/Epidyolex (>99% CBD), and three synthetic cannabis-related drug products: Marinol and Syndros (dronabinol (–),-Δ^9^-trans-tetrahydrocannabinol) for nausea associated with cancer chemotherapy and for the treatment of anorexia associated with weight loss in AIDS patients, and Cesamet (nabilone, THC analog) indicated for nausea associated with cancer chemotherapy, usually after previous treatments have failed.

The European Medicines Agency (EMA) approved to the date Sativex (Nabiximols, cannabidiol/delta-9-tetrahydrocannabinol) for the treatment of chronic pain in palliative care in children when optimal treatment with opiates is not fully effective, and in patients with multiple sclerosis (MS) who have not responded sufficiently to other antispasticity medication. Following the FDA, EMA approved Epidiolex (165) dronabinol and nabilone (166). Nonetheless, the majority of countries have their own legal regulations regarding the medical use of cannabinoids, and only a few have established special access schemes to allow cannabis preparations for the treatment of a narrow range of medical conditions.

It seems inevitable that research will be directed towards finding better synthetic cannabinoids having full agonistic activity and increased affinity to both CB1 and CB2 receptors and higher therapeutic window as well. However, the rapidly increasing number of reported intoxication and acute failures must be taken into specific consideration and special emphasis given to further research and registration of these compounds (167).

## Summary and Future Outlook

Crohn’s disease and ulcerative colitis are the two primary forms of inflammatory bowel disease (IBD): chronic, relapsing-remitting, or progressive inflammatory condition of the gastrointestinal tract. IBD is an extremely complex disease with an incompletely understood pathogenesis. The endocannabinoid system (ECS) is involved in intestinal homeostasis, modulation of gastrointestinal motility, intestinal barrier integrity, visceral sensing, and the regulation of inflammation. ECS is a crucial player in dampening cytokine production and in inhibiting leukocyte adhesion and activation. With an in-depth understanding of the endocannabinoid system’s role, increasing evidence suggests a close relationship between gastrointestinal diseases and the endocannabinoid system homeostasis disturbances, suggesting that the ECS system may be an excellent future pharmacological target.

To date, many components of the endocannabinoid system have been suggested as potential targets for drug therapy. However, due to the relative paucity of preclinical evidence describing a beneficial role for the ECS in the treatment of gastrointestinal diseases in humans, the often varying and ambiguous expression levels of the ECS system compounds depending on the disease entity and the multitude of mediators associated with endocannabinoids, makes the matter quite problematic. We now have extensive evidence from experimental models of inflammatory bowel disease on the role of cannabinoid agonists and inhibitors of endocannabinoid degradation in rodent models of IBD, showing the potential therapeutic potential of cannabinoids. Studies described so far in the literature using MGL (108), CB1 and CB2 receptor agonists (24, 26, 28), GPR55 antagonists (90), FAAH inhibitors (168), phytocannabinoids, including cannabidiol and cannabigerol (51, 169) showed protection against experimental inflammation of the gastrointestinal tract. Interestingly, these studies show that the phytocannabinoids action mechanism is also through novel components of the endocannabinoidome rather than *via* the cannabinoid receptors solely. Therefore, a further direction should be to unravel the role of less studied components of the ECS, such as orphan G-protein-coupled receptors including GPR40, GPR41, GPR43, GPR55, GPR84, GPR119, and GPR120. It also appears that treatment with broadly defined endocannabinoidome components such as palmitoylethanolamide (106), endogenous cannabinoid receptor agonists: anandamide (112) and MGL inhibitors (108) has been shown to reduce intestinal inflammation and associated systemic and central inflammation very efficiently. This observation demonstrates that gastrointestinal inflammation can be reduced by exo- and endogenous cannabinoids. Cannabinoids inhibit inflammation under physiological and pathophysiological conditions and consequently relieve disease symptoms. This property of cannabinoids is mediated through multiple pathways, such as inducing apoptosis in activated immune cells or suppressing cytokines and chemokines at inflammatory sites. Determining the relationship between ECS and disease activity, severity and phenotype would provide the opportunity to use markers of the endocannabinoid system for diagnosis and monitoring of patients with IBD. The development of new therapies with high efficacy and minimal side effects is becoming an important goal of IBD research. Therefore, conducting translational research to understand the role of the endocannabinoid system in the pathogenetic mechanisms of human IBD remains crucial for the effective treatment of these multifactorial diseases.

## Data Availability Statement

The original contributions presented in the study are included in the article. Further inquiries can be directed to the corresponding author.

## Author Contributions

All authors approved the submitted version and have made substantial contributions to all of the following: (1) the conception and design of the study, or acquisition of data, or analysis and interpretation of data, (2) drafting the article or revising it critically for important intellectual content, (3) final approval of the version to be submitted. SH conceptually designed the review. SH, MK-R searched the literature, prepared the table, and wrote the manuscript. AZ searched the literature, prepared the figure and wrote the manuscript. SH, MKR, RJS and AP provided intellectual input and edited the manuscript. RS provided intellectual input, participated in the conceptual design of the review. The Authors had no writing assistance while drafting the article.

## Conflict of Interest

The authors declare that the research was conducted in the absence of any commercial or financial relationships that could be construed as a potential conflict of interest.

## Publisher’s Note

All claims expressed in this article are solely those of the authors and do not necessarily represent those of their affiliated organizations, or those of the publisher, the editors and the reviewers. Any product that may be evaluated in this article, or claim that may be made by its manufacturer, is not guaranteed or endorsed by the publisher.

## Abbreviations

IBD inflammatory bowel disease**;** GI gastrointestinal tract; CD Crohn’s disease; UC ulcerative colitis; ECS Endocannabinoid system; ROS reactive oxygen species; CB1 and CB2 cannabinoid receptors type 1 and type 2; AEA anandamide; 2-AG Arachidonoylglycerol; DAGL Diacylglycerol lipase; NAPE-PLD *N*-acylphosphatidyl-ethanolamine phospholipase; FAAH Fatty acid amide hydrolase; MGL Monoacylglycerol lipase; OEA Oleoylethanolamide; PEA Palmitoylethanolamide; COX2 Cyclooxygenase-2; IL-1β Interleukin 1 beta; DSS Dextran sulfate sodium; DNBS Dinitrobenzene sulfonic acid; VEGF-A Vascular endothelial growth factor A; TNF-α Tumor necrosis factor alpha; jwh133 Potent selective CB2 receptor agonist; IL-12:Interleukin 12; IL-6 Interleukin 6; HU-210 Synthetic cannabinoid**;** WIN 55, 212-2 Potent cannabioid receptor agonist; CXCL1/KC Keratinocyte-derived chemokine; ICAM-1 Intercellular Adhesion Molecule 1; VCAM-1 Vascular cell adhesion protein 1; PECAM-1 Platelet endothelial cell adhesion molecule; GPR G protein-coupled receptors; GLP-2 Glucagon-like peptide-2; TRP Transient receptor potential channel; 5HT3 5-hydroxytryptamine or serotonin receptors; PPAR- α, γ, and δ peroxisome proliferator-activated receptors alpha, betta and gamma; AA arachidonic acid; Th1 T helper 1 cell; Th17 T helper 17 cell; CX3CR1 chemokine receptor 1, Mϕ macrophages; MAEA methanandamide; EMA European Medicines Agency; JZL184 irreversible inhibitor for monoacylglycerol lipase; SR141716A selective cannabinoid receptor 1 antagonist; AM630 6-Iodopravadoline, cannabinoid receptor CB2 potent and selective inverse agonist; AM1241 cannabinoid CB2 receptor selective agonist; CDAI Crohn’s Disease Activity Index; CBD Cannabidiol; THC delta-9-tetrahydrocannabinol.

## Glossary

**Table:**

IBD	inflammatory bowel disease
GI	gastrointestinal tract
CD	Crohn’s disease
UC	ulcerative colitis
ECS	Endocannabinoid system
ROS	reactive oxygen species
CB1 and CB2	cannabinoid receptors type 1 and type 2
AEA	anandamide
2-AG	Arachidonoylglycerol
DAGL	Diacylglycerol lipase
NAPE-PLD	*N*-acylphosphatidyl-ethanolamine phospholipase
FAAH	Fatty acid amide hydrolase
MGL	Monoacylglycerol lipase
OEA	Oleoylethanolamide
PEA	Palmitoylethanolamide
COX2	Cyclooxygenase-2
IL-1β	Interleukin 1 beta
DSS	Dextran sulfate sodium
DNBS	Dinitrobenzene sulfonic acid
VEGF-A	Vascular endothelial growth factor A
TNF-α	Tumor necrosis factor alpha
jwh133	Potent selective CB2 receptor agonist
IL-12	Interleukin 12
IL-6	Interleukin 6
HU-210	Synthetic cannabinoid
WIN 55, 212-2	Potent cannabioid receptor agonist
CXCL1/KC	Keratinocyte-derived chemokine
ICAM-1	Intercellular Adhesion Molecule 1
VCAM-1	Vascular cell adhesion protein 1
PECAM-1	Platelet endothelial cell adhesion molecule
GPR	G protein-coupled receptors
GLP-2	Glucagon-like peptide-2
TRP	Transient receptor potential channel
5HT3	5-hydroxytryptamine or serotonin receptors
PPAR- α, γ, and δ	peroxisome proliferator-activated receptors alpha, betta and gamma
AA	arachidonic acid
Th1	T helper 1 cell
Th17	T helper 17 cell
CX3CR1	chemokine receptor 1
Mϕ	macrophages
MAEA	methanandamide
EMA	European Medicines Agency
JZL184	irreversible inhibitor for monoacylglycerol lipase
SR141716A	selective cannabinoid receptor 1 antagonist
AM630	6-Iodopravadoline, cannabinoid receptor CB2 potent and selective inverse agonist
AM1241	cannabinoid CB2 receptor selective agonist
CDAI	Crohn’s Disease Activity Index
CBD	Cannabidiol
THC	delta-9-tetrahydrocannabinol
